# Land–air coupling over the Tibetan Plateau and its climate impacts

**DOI:** 10.1093/nsr/nwaa012

**Published:** 2020-02-06

**Authors:** Guoxiong Wu

**Affiliations:** 1 Professor, State Key Laboratory of Numerical Modeling for Atmospheric Sciences and Geophysical Fluid Dynamics, Institute of Atmospheric Physics, Chinese Academy of Sciences, China; 2 Guest Editor of Special Topic

The mechanical and thermal effects of the gigantic Tibetan Plateau (TP), together with its unique *in situ* land–air coupling and troposphere–stratosphere interaction, play an important role in the atmospheric general circulation, regional weather and climate, variability of the Asian summer monsoon and the global atmospheric energy and water cycle. As evidenced by the striking warming trend, the climate and atmospheric energy budget over the TP has changed more significantly than that averaged over the rest of the globe in the past three decades. The climate impact of the TP has therefore become a scientific frontier in global climate-change studies. However, due to scarce observations, bias in circulation models and lack of physical understanding, our knowledge of energy and water exchanges over the TP and their climate impacts is still limited.

In the face of these great challenges, a 10-year research program called ‘Land-Air coupled System over the Tibetan Plateau and its Impacts on global Climate’ was launched by the National Natural Science Foundation of China in 2013. Its core scientific goals include further understanding of land–air coupling processes, development of a land–air data-assimilation system in the TP domain, improvement in regional and global climate models, and revealing the mechanism of TP weather and climate impacts. This Special Topic is devoted to giving a timely review of the recent progress in the program and providing new perspectives on its future direction.

This Special Topic includes three reviews. The first, by Fu *et al.*, concerns the progress of observations of the atmospheric boundary layer, land-surface heat fluxes and cloud-precipitation distribution and vertical structure by using ground- and space-based multiplatforms, as well as the effect of cloud systems in the TP on downstream weather. The second, by Bian *et al.*, presents a review of major recent progress in research regarding the cross-tropopause exchange of pollutants in the TP region based on satellite observations, *in situ* measurements and model simulations. It summarizes how, in the summer, surface emissions and ozone-poor air are transported upward to the upper troposphere and into the South Asian High to produce enhanced pollutants and low ozone in the upper troposphere and lower stratosphere over the TP, and how the significant Asian Tropopause Aerosol Layer is generated. Suggestions for further studies are also proposed. The third, by Liu YM *et al.*, reviews recent advances in the dynamics of TP climate. It shows that thermal forcing over the TP together with the Iranian Plateau forms a coupled system whose heating generates a monsoonal meridional circulation and creates a favorable background for the development of the Asian summer monsoon. The TP also exerts strong impacts on upstream climate variations and significantly affects oceanic circulation and buoyancy fields, contributing to the formation of the Atlantic Meridional Overturning Circulation. Moreover, the variability of the Asian summer monsoon is controlled synergistically by the TP thermal state and the atmosphere–ocean interaction.

Accompanying the reviews are three Perspectives focusing on the arid climate in northwestern China as it relates to TP forcing (Liu YZ *et al.*), aerosol characteristics over the TP and its local impacts on weather and climate (Zhao *et al.*), and land–air data assimilation in the TP (Yang *et al.*). This Special Topic also highlights recent progress on the development of a high-resolution climate-system model for more accurate TP-climate modeling (Bao and Li) and the unique characteristics of lightning activity and its relationship with convection over the TP (Zheng *et al.*). Several intriguing and cutting-edge issues for future studies are also proposed in this Special Topic.

At the end, an interview with Prof. Soroosh Sorooshinan, a distinguished world leader in land–air interaction research, is presented, which provides profound insights on land–air interaction over high terrain and advice for future studies of global land–air–sea interactions. As a final note, I would like to thank all authors, editorial staff, international and domestic reviewers and everyone else who has contributed to the initiation and completion of this Special Topic. Special acknowledgment is due to Professors Xiuji Zhou, Xiangde Xu, Jianping Huang and Anmin Duan and to Dr. Chaolin Zhang for their cooperation.

